# Supplementary figures and images for: Flexibility and intrinsic disorder are conserved features of hepatitis C virus E2 glycoprotein
Source: PLoS Comput Biol. 2020 Feb 28;16(2):e1007710. doi: 10.1371/journal.pcbi.1007710 (PMC7065822; doi:10.1371/journal.pcbi.1007710)

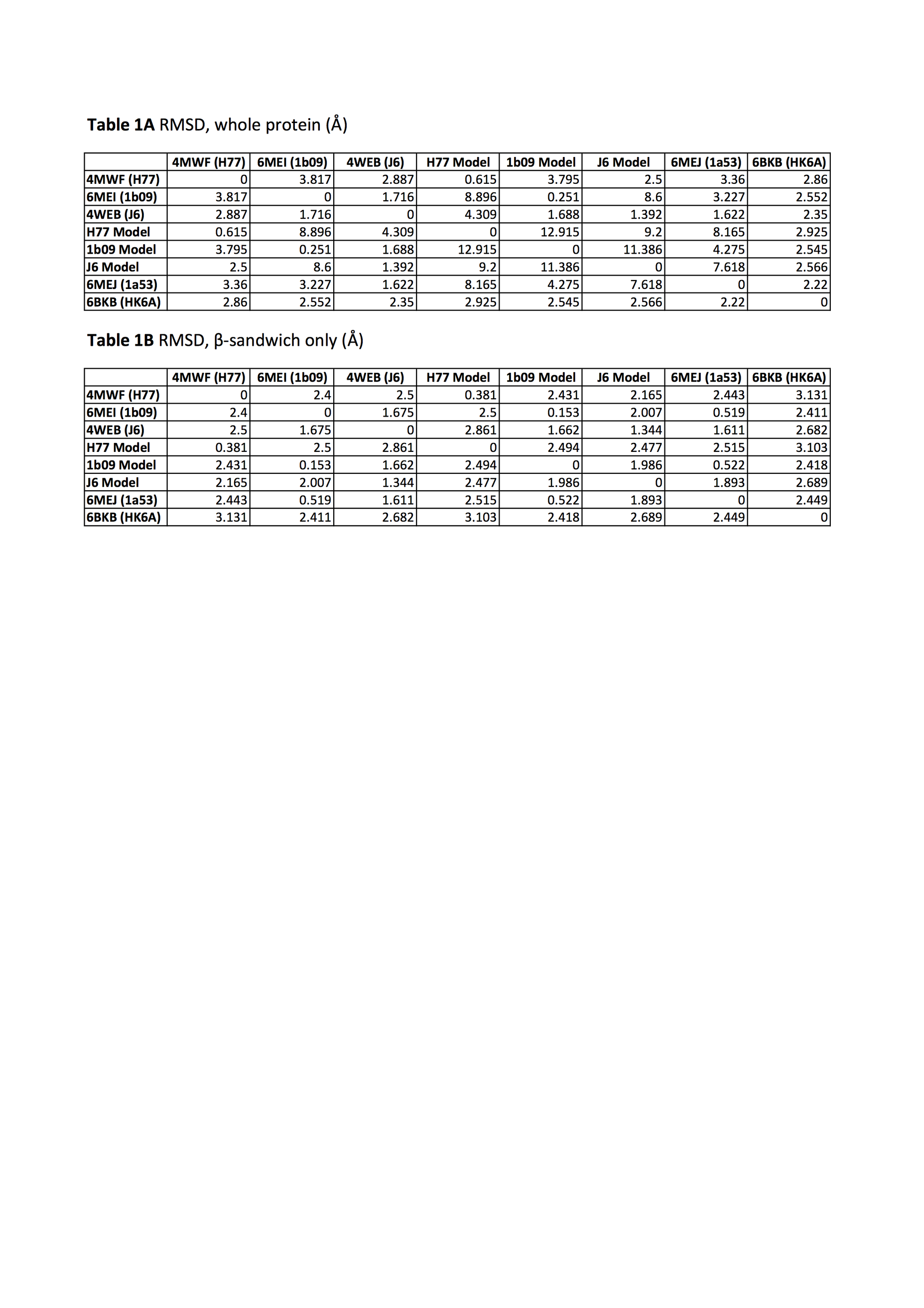

Supplement: S1 Table — A. Pairwise backbone RMSD comparison of each E2 model with its parent crystal structure and with E2 from alternative strains (1a53 and HK6A). B. As above, but with the analysis limited to the β-sandwich; this provides a degree of agreement between an unmodelled portion of the protein. (TIF) [file pcbi.1007710.s001.tif]

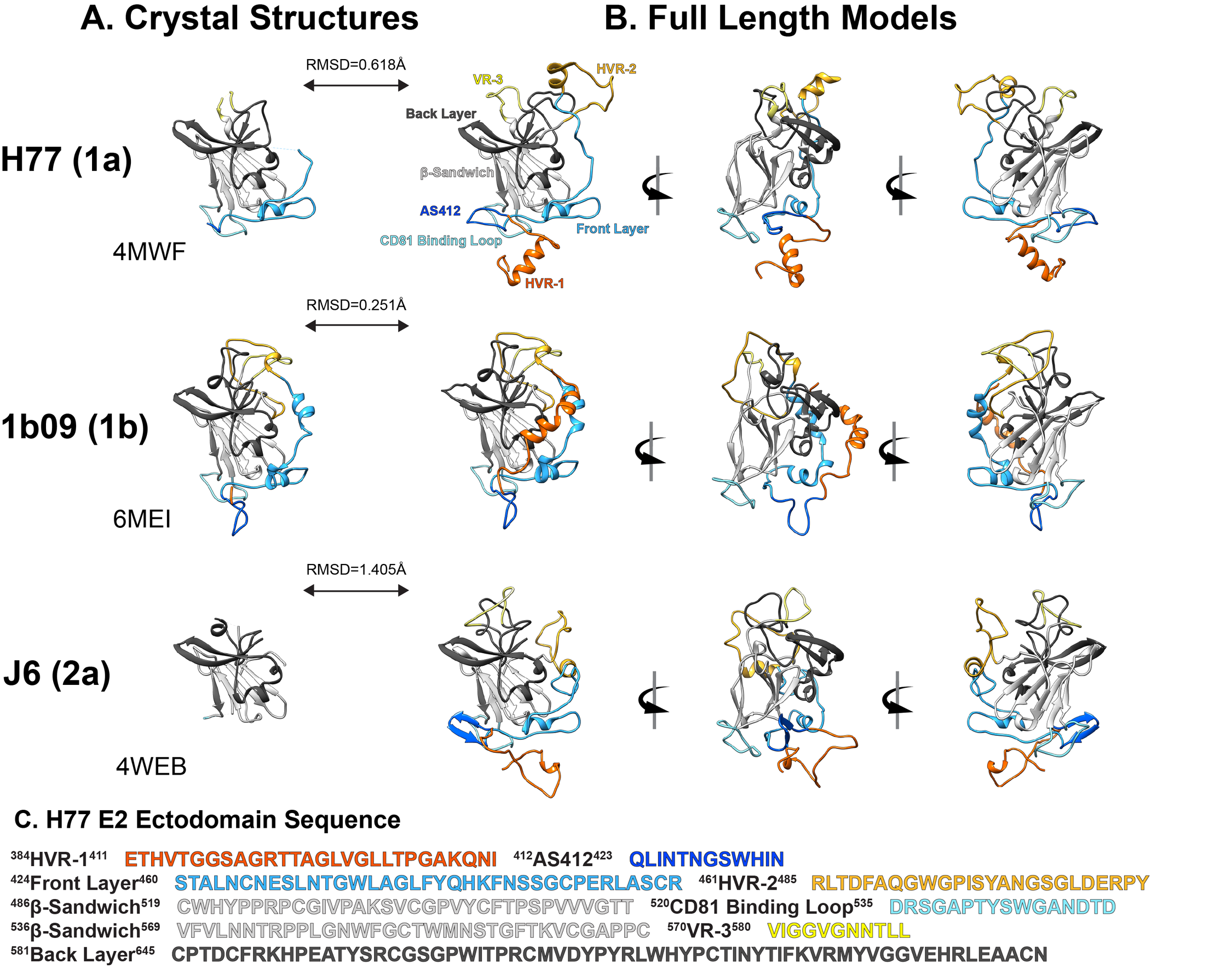

Supplement: S1 Fig — Partial crystal structures were used as the basis for building full length models of the E2 ectodomain A. Partial crystal structures of E2 from H77 (PDB 4MWF), 1b09 (PDB 6MEI) and J6 (PDB 4WEB). B. Corresponding complete E2 ectodomain models shown at 0°, 90° and 180° rotation. Backbone RMSD values reflect divergence between crystal structures and models; all RMSD values are low, indicating good agreement. C. H77 E2 ectodomain sequence organised by protein region. Numbering (relative to start of HCV polyprotein) defines region assignments. (TIF) [file pcbi.1007710.s002.tif]

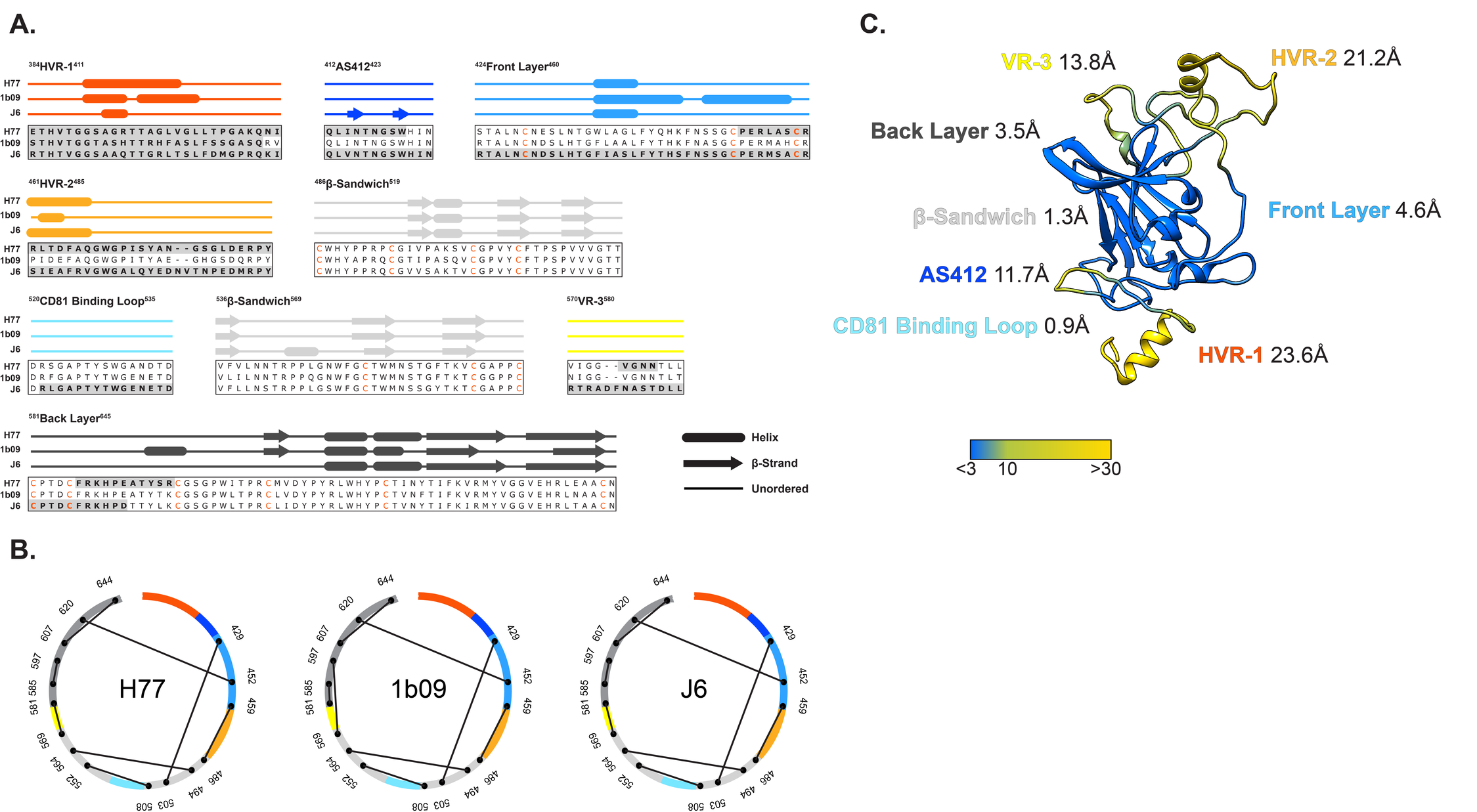

Supplement: S2 Fig — A. Aligned protein sequence from each model, organised by region, modelled regions are shaded in grey and conserved cysteine residues are orange. Secondary structure assignments are shown above the sequences. B. Disulphide bonding pattern for each model, cysteine positions are numbered according to the H77 reference sequence. C. H77 ectodomain model color coded to display backbone RMSD between models; region names are annotated with their average RMSD value (the mean RMSD of all residues within a given region). High RMSD values indicate disagreement between the models. For RMSD analysis, model structures were aligned using the β-Sandwich as a reference. (TIF) [file pcbi.1007710.s003.tif]

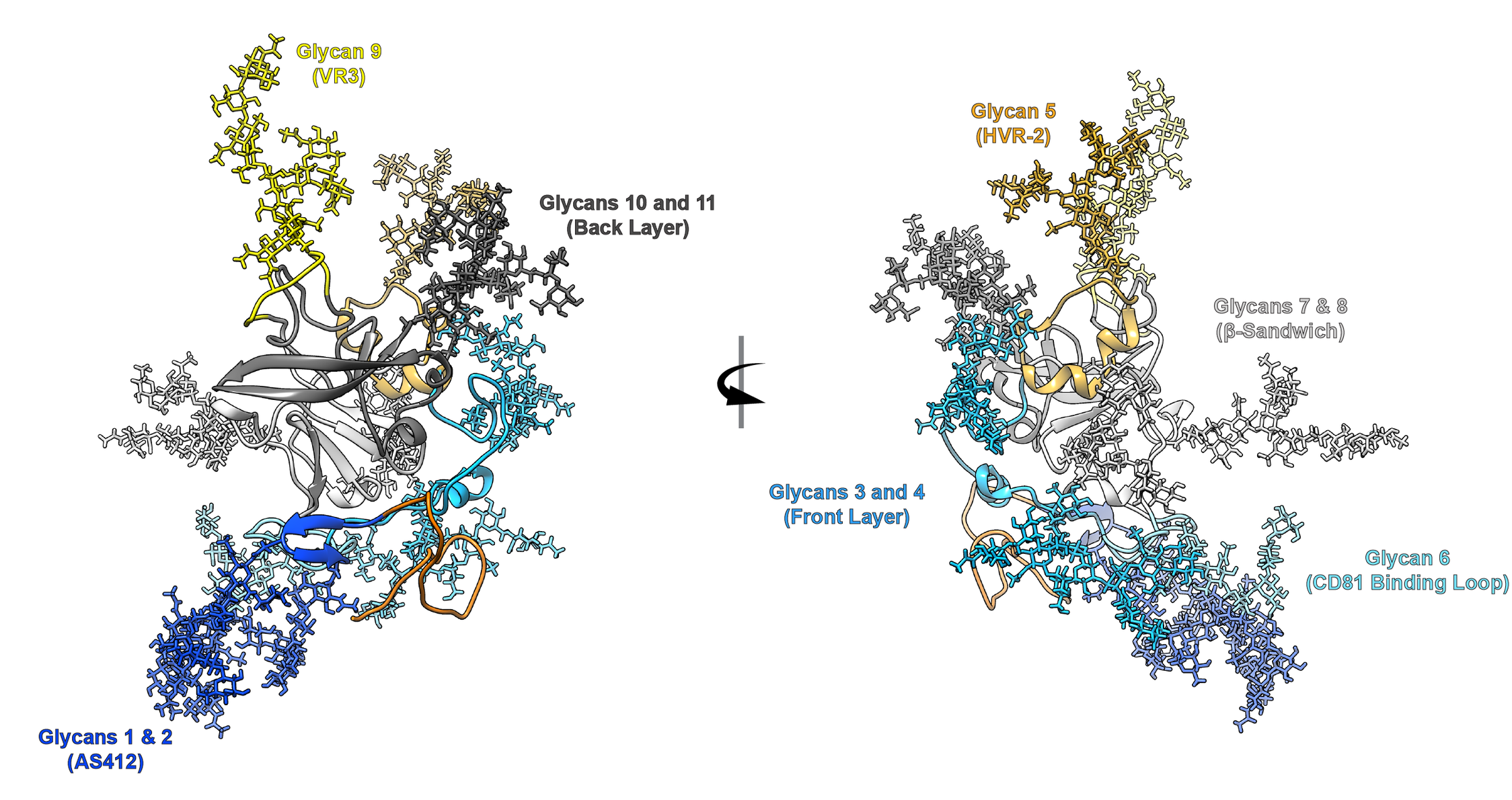

Supplement: S3 Fig — Glycans are color coded according to protein region. Models are shown at 0° and 200° rotation. (TIF) [file pcbi.1007710.s004.tif]

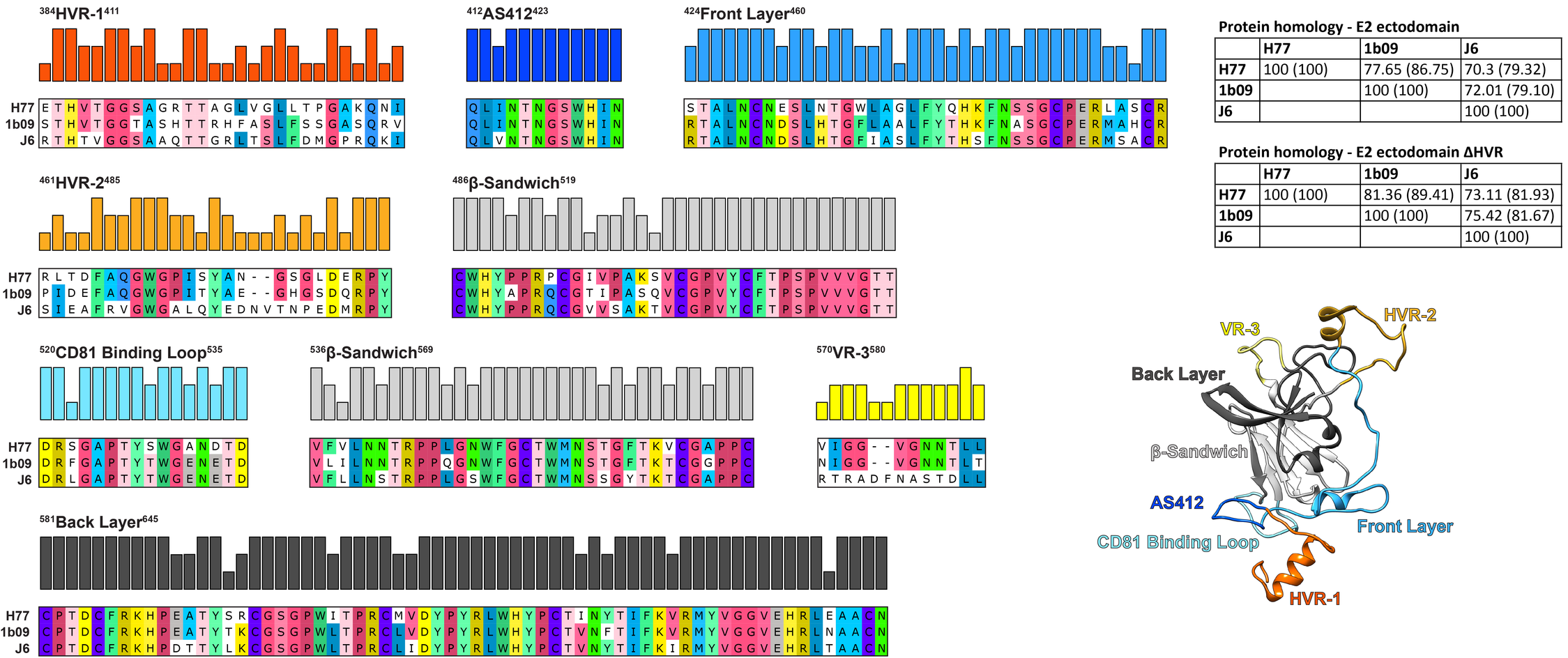

Supplement: S4 Fig — Protein sequence is organised by region. Bars indicate level of conservation at each position. Tables indicate pairwise protein homology for the entire E2 ectodomain and without HVR-1, which is the major source of divergence. Values represent % identity and % similarities in parentheses (taking into account the equivalencies of certain amino acids). (TIF) [file pcbi.1007710.s005.tif]

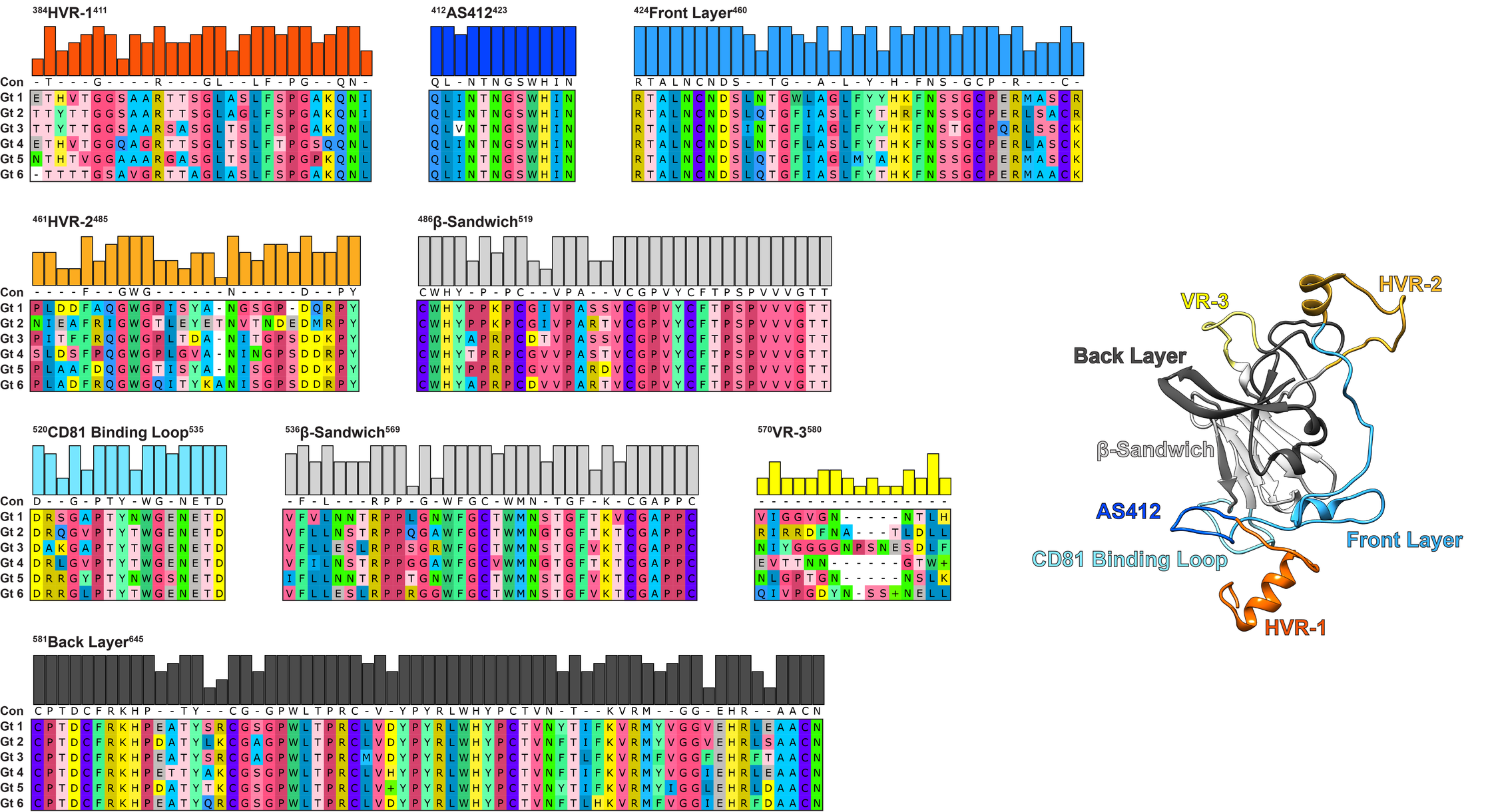

Supplement: S5 Fig — Aligned consensus E2 sequences from HCV genotypes 1–6, organised by protein region. Bars indicate level of conservation at each position. The consensus sequence (Con) indicates residues that are conserved in all 6 sequences. (TIF) [file pcbi.1007710.s006.tif]

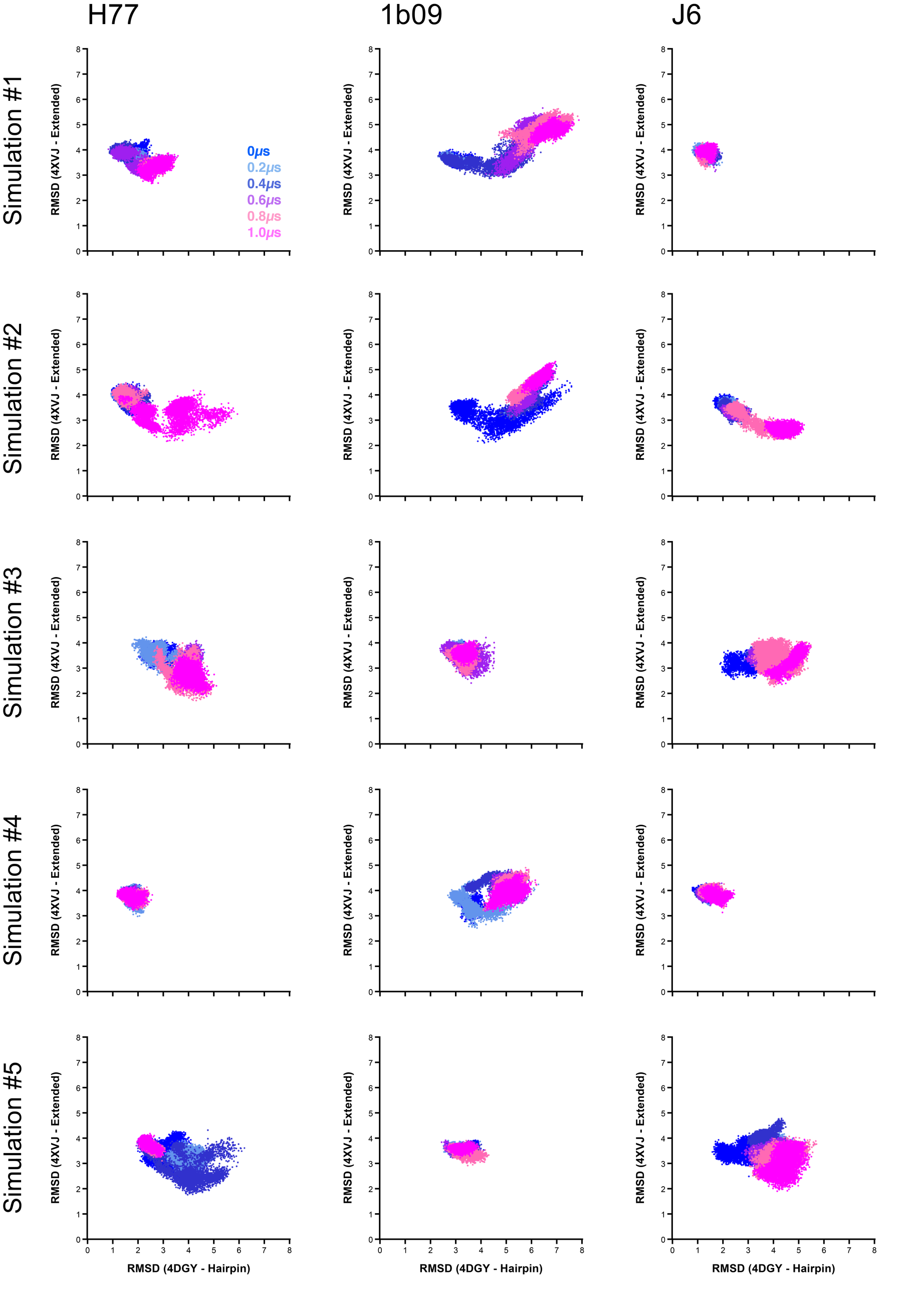

Supplement: S6 Fig — Scatter plots of backbone RMSD values between each MD trajectory and reference structures in the β-hairpin (PDB 4DGY) or extended (PDB 4XVJ) conformations. The data points represent individual frames and are color-coded by time, as stated in the legend. (TIF) [file pcbi.1007710.s007.tif]

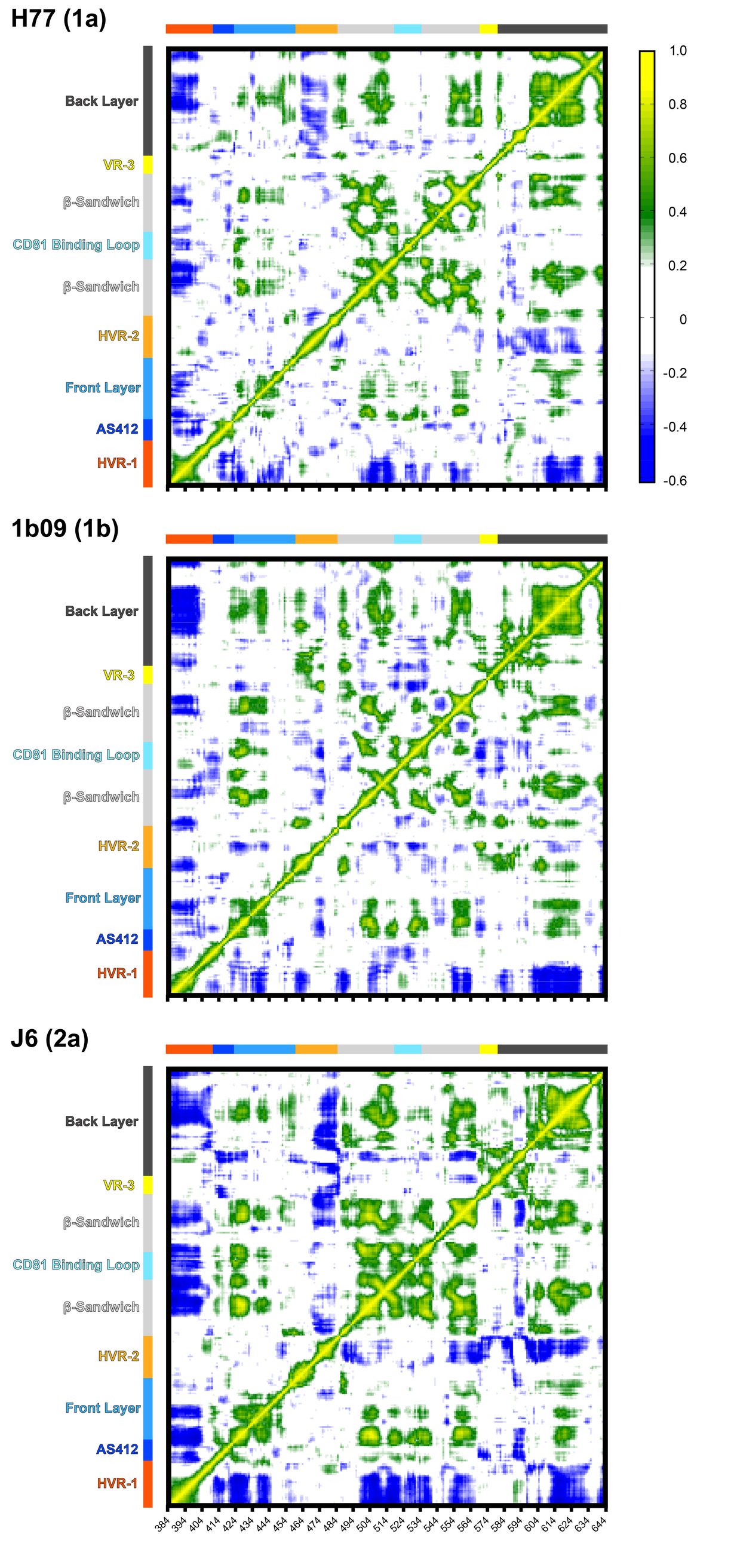

Supplement: S7 Fig — DCC provides a residue-by-residue pairwise comparison of motion in MD trajectories to reveal correlations/anti-correlations in protein movement. DCC analysis was performed on MD data from H77, 1b09 and J6. Color-coding indicates the degree of correlation. (TIF) [file pcbi.1007710.s008.tif]

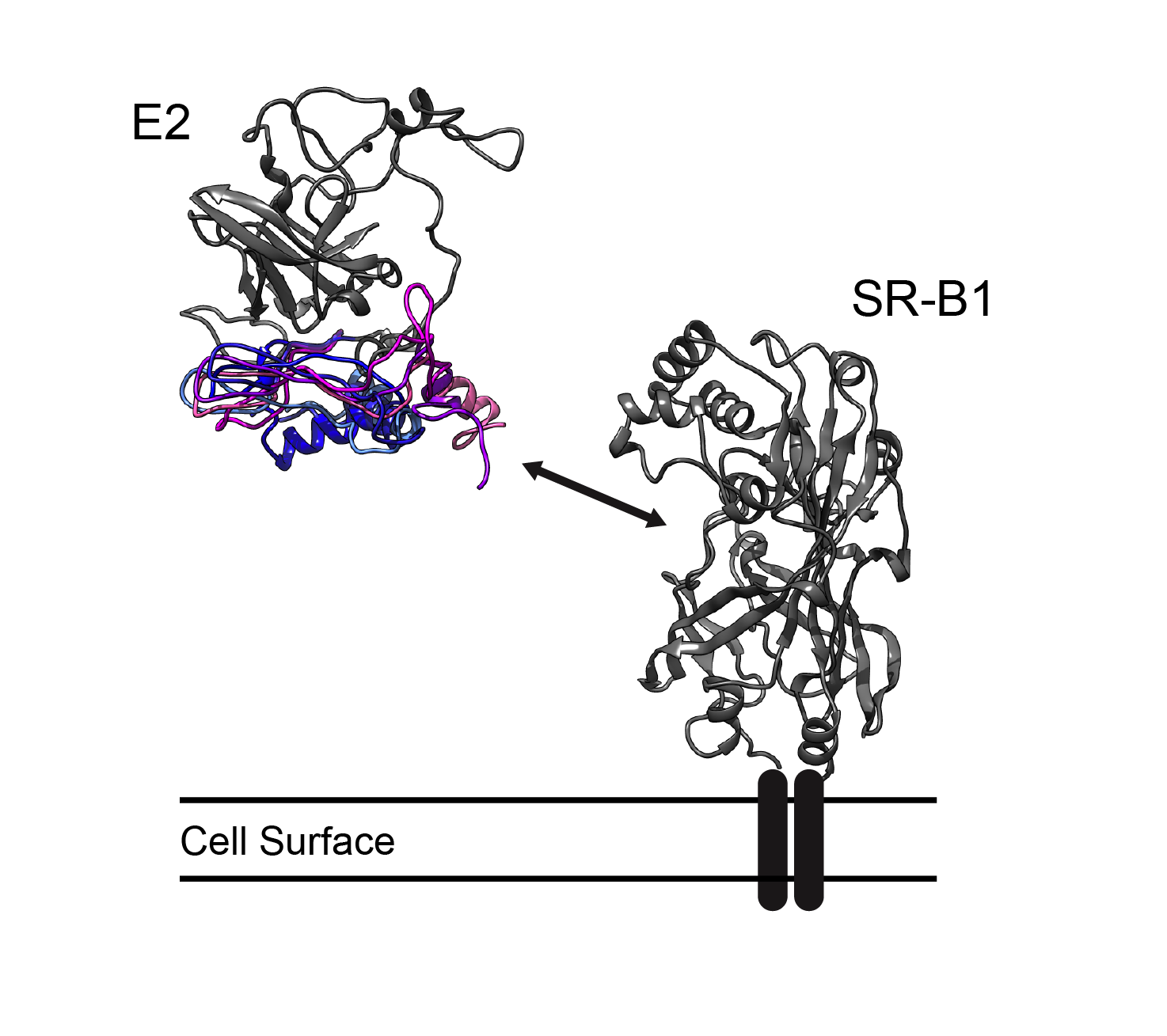

Supplement: S8 Fig — SR-B1 is a receptor for HCV that interacts with E2 via HVR-1. Therefore, it is likely that the flexible and largely disordered HVR-1 will become constrained upon interaction. This may provide a mechanism by which receptor binding is communicated to the rest of E2. Image depicts H77 E2 with alternative conformations of HVR-1 (color coded by time, as in Fig 3) and a homology model of SR-B1 based on the structure of LIMP-2 (PDB 4F7B). (TIF) [file pcbi.1007710.s009.tif]
