# Supplementary material for: Flexibility and intrinsic disorder are conserved features of hepatitis C virus E2 glycoprotein
Source: PLoS Comput Biol. 2020 Feb 28;16(2):e1007710. doi: 10.1371/journal.pcbi.1007710 (PMC7065822; doi:10.1371/journal.pcbi.1007710)
Supplement: S4 File — Modeller and Rosetta software scripts used to create E2 models. (DOCX) [file pcbi.1007710.s013.docx]

1. **Modeller Files and Commands**
2. **Rosetta Relax Files and Commands**
3. **Rosetta HVR2 loop Files and Commands**
4. **Rosetta N-terminal HVR1 Files and Commands**
5. **Modeller Files and Commands**

This script builds the initial 1000 structures and accesses them with the DOPE score. The alignment and the known sequence was altered for each HCV strain that we modelled according to the specific structure (e.g. in this case it is PDB 4MWF). The number of residues in the self.patch command refer to the required disulphide bond in the HVR2 loop and was changed accordingly. The numbering starts with the number 1 as opposed to number 384.

from modeller import *

from modeller.automodel import *

log.verbose()

class MyModel(automodel):

def special_patches(self, aln):

self.patch(residue_type='DISU', residues=(self.residues['76'], self.residues['103']))

env = environ()

env.io.atom_files_directory = ['.', '../atom_files']

# Build models, and assess with DOPE

a = MyModel(env, alnfile = 'alignment_full.ali',

knowns = (‘4mwf_chain_D.pdb','4dgv_A.pdb','4dgy_A.pdb','4g6a_A.pdb','4gag_p.pdb','4gaj_P.pdb','4hs6_Z.pdb','4wht_i.pdb','5eoc_q.pdb','4hs8_A.pdb'),

sequence = '4mwf_d.full',

assess_methods=(assess.DOPE)) # assess loops with DOPE

a.md_level = refine.slow

a.starting_model= 1

a.ending_model = 1000

a.make()

This script refines the loop region between 574 - 577 and 586 – 596 from the lowest scoring model of the initial build. The numbering again starts at 1 and will change, together with the sequence, according to the structure used.

# Loop refinement of an existing model

from modeller import *

from modeller.automodel import *

log.verbose()

env = environ()

# directories for input atom files

env.io.atom_files_directory = ['.', '../atom_files']

# Create a new class based on 'loopmodel' so that we can redefine

# select_loop_atoms

class MyLoop(loopmodel):

# This routine picks the residues to be refined by loop modelling

def select_loop_atoms(self):

return selection(self.residue_range('203:', '213:'),

self.residue_range('191:', '194:'))

a = MyLoop(env,

inimodel=‘input_model.pdb’, # initial model of the target

sequence='4mwf_d.full',

loop_assess_methods=assess.DOPE) # assess loops with DOPE

a.loop.starting_model= 1

a.loop.ending_model = 1000

a.loop.md_level = refine.slow

a.make()

Both scripts were run using the command: python filename.py

1. **Rosetta Relax Files and Commands**

This script was used to relax the best scoring structure generated by the Modeller scripts using this shell script:

#!/bin/bash

if [ $# -ne 1 ]; then

echo "Usage: $0 pdb_file"

exit 1

fi

rosetta="/s/rosetta/v3.7/rosetta_bin_linux_2016.32.58837_bundle/main/source/bin/relax.linuxgccrelease"

database="/s/rosetta/v3.7/rosetta_bin_linux_2016.32.58837_bundle/main/database"

pdb=$1

seed=12

$rosetta -database $database -s $pdb @flags.relax

With the command: ./relax_script.sh input.pdb

The content of the @flags.relax file:

-ignore_unrecognized_res

-relax:constrain_relax_to_start_coords

-relax:ramp_constraints false

-ex1

-ex2

-use_input_sc

-correct

-no_his_his_pairE

-no_optH false

-flip_HNQ

-renumber_pdb F

-overwrite

-nstruct 1

1. **Rosetta HVR2 loop Files and Commands**

This script was used to model the HVR2 loop using the relaxed structure using this shell script:

#!/bin/bash

if [ $# -ne 3 ]; then

echo "Usage: $0 pdb_file prefix seed"

exit 1

fi

rosetta="/s/rosetta/v3.7/rosetta_bin_linux_2016.32.58837_bundle/main/source/bin/loopmodel.linuxgccrelease"

database="/s/rosetta/v3.7/rosetta_bin_linux_2016.32.58837_bundle/main/database"

pdb=$1

prefix=$2

seed=$3

$rosetta -database $database -s $pdb -out:prefix $prefix -jran $seed @flags.loop

With the command: ./HVR2loop_script.sh input.pdb aa 12

Where the “aa” stands for a prefix and “12” is the random seed. Both of these parameters need to change with each run.

The content of the @flags.loop file:

-in:ignore_unrecognized_res

-overwrite

-nstruct 200

-constant_seed

-ex1

-ex2

-loops:remodel perturb_kic_with_fragments

-loops:refine refine_kic_with_fragments

-loops:fix_natsc

-rebuild_disulf true

-loops:loop_file Loop.loops

-constraints:cst_fa_file loop.fa.cst

-constraints:cst_fa_weight 1.0

-constraints:cst_file loop.cst

-constraints:cst_weight 1.0

-loops:frag_sizes 9 3

-loops:frag_files /d/as12/u/sl002/rosetta/run/loop/run_fr_1/fragments_9.txt /d/as12/u/sl002/rosetta/run/loop/run_fr_1/fragments_3.txt

-in:file:fullatom

-out:file:fullatom

The contents of the Loops.loops, loop.fa.cst and loop.cst files will need to change according to the modelled strain.

Loop.loops content:

LOOP 70 108

Here, we specify the residue range of the loop to be modelled. The numbering starts from 1.

loop.fa.cst content:

AtomPair SG 76 SG 103 HARMONIC 2.05 0.05

In this file, we specify the sulphur atoms forming the disulfide bond in the HVR2.

loop.cst content:

AtomPair CA 76 CA 103 HARMONIC 5.0 1.0

In this file, we specify the alpha carbon atoms forming the disulfide bond in the HVR2.

1. **Rosetta N-terminal HVR1 Files and Commands**

This script was then used to model the N-terminal HVR1 using this shell script:

#!/bin/bash

if [ $# -ne 3 ]; then

echo "Usage: $0 pdb_file prefix seed"

exit 1

fi

rosetta="/s/rosetta/v3.7/rosetta_bin_linux_2016.32.58837_bundle/main/source/bin/FloppyTail.linuxgccrelease"

database="/s/rosetta/v3.7/rosetta_bin_linux_2016.32.58837_bundle/main/database"

pdb=$1

prefix=$2

seed=$3

$rosetta -database $database -s $pdb -out:prefix $prefix -jran $seed @flags.HVR1

With the command: ./HVR1_script.sh input.pdb aa 12

Where the “aa” again stands for a prefix and “12” is the random seed. Both of these parameters need to change with each run.

The content of the @flags.HVR1 file:

-ignore_unrecognized_res

-ex1

-ex2

-use_input_sc

-constant_seed

-packing:repack_only

-run:min_type dfpmin_armijo_nonmonotone

-FloppyTail:flexible_chain D

-flexible_start_resnum 1

-flexible_stop_resnum 29

-flexible_chain D

-short_tail_off 0.2

-short_tail_fraction 0.33

-shear_on 0.333333333333333333

-FloppyTail:refine_repack_cycles 40

-FloppyTail:perturb_cycles 5000

-FloppyTail:refine_cycles 3000

-FloppyTail:refine_temp 0.8

-FloppyTail:perturb_temp 0.8

-C_root

-in::file::frag3 fragment_3.txt

-in::file::frag9 fragmets_9.txt

-overwrite

-nstruct 200

Here, we can change the number of the start and stop residue.
