# Supplementary material for: Flexibility and intrinsic disorder are conserved features of hepatitis C virus E2 glycoprotein
Source: PLoS Comput Biol. 2020 Feb 28;16(2):e1007710. doi: 10.1371/journal.pcbi.1007710 (PMC7065822; doi:10.1371/journal.pcbi.1007710)
Supplement: Attachment — Submitted filename: Detailed Response to Reviewers.docx [file pcbi.1007710.s021.docx]

Below we have listed the verbatim reviewers comments and our responses. Note that line number references refer to the marked up copy of the manuscript.

**Reviewer #1:** The authors provide an in-depth study of the dynamics of the HCV E2 ectodomain, relating it to key sequence features, as well as bnAb and coreceptor engagement. While this provides new insights into HCV E2 and its function, there are some issues which may limit the accuracy of the modeling, including lack of E1 glycoprotein in the models, which interacts with E2 in the native virion, likely at some E2 sites that are considered in this study. Given the lack of an experimentally determined E1-E2 co-crystal structure, this was a necessary omission, and the authors do note this shortcoming in the Discussion. Overall, this study is a useful addition to the field, but several comments should be addressed by the authors to improve the clarity and to support the conclusions of this study:

**Major comments:**

1. It is concerning that there does not seem to be a way readily to gauge the accuracy of the molecular dynamics simulations and the readout, including the RMSF information (Figs. 2 and 3), to ensure that the modeling represents true E2 dynamic behavior on the virion. The authors could, for instance, assess (based on backbone RMSD) whether sampled or preferred states of AS412 from MD simulations recapitulate the states observed in x-ray structures of that epitope engaged by bnAbs HCV1 and HC33.1, or alternatively they can assess against the x-ray structure of the AS434 epitope engaged by bnAb HC84.1. It is possible that lack of preference for these conformations in the MD simulations could point to “induced fit” behavior, rather than a shortcoming of the simulations themselves. At very least, the authors should make a brief note of this uncertainty, if they are unable to directly demonstrate through correspondence with experimental structural or other data that the MD simulations are accurate representations of native E2.

We thank the reviewer for this excellent suggestion. We completed further analysis of our data, performing quantitative comparison of each MD trajectory to the suggested AS412 crystal structures. This proved to be very informative and demonstrated that our MD experiments recapitulate the conformational transitions evident in published structures. This greatly enhances this study; both validating our experiments and providing insights in to the conformational dynamics of this functionally important region of E2.

This new material is provided as a new Figure 5 (and associated S6 Fig); it is integrated in to the text at line 267 and line 521.

2. For the consensus sequence generation, it is not clear whether the authors performed any analysis or filtering to avoid redundancy or over-representation of certain sequences or subtypes, e.g. by culling E2 sequences with >98% identity to any others in the dataset. This would reduce the effect of any sampling bias in the HCV sequence databases. Such methods should be noted explicitly by the authors. If no redundancy filter or criterion was used, the authors should consider using one, or they should at very least note why one would not be needed.

Thank you for pointing this out. We performed 98% filtering of our alignments to remove redundant sequences. This resulted in subtle revisions to multiple figures but did not alter any of our results or conclusions.

Revised versions of Fig 4, S5 Fig, Fig 6 (previously Fig 5) and Fig 7 (previously 6) are provided. The amended methods are detailed at line 770.

3. To further investigate sequence diversity and structural/dynamic conservation, it would be reasonable to include one of the genotype 6 E2 core x-ray structures reported by Tzarum et al (reference 13), which were released in the Protein Data Bank late last year. It is possible that the relatively recent release of these structures prevented analysis by the authors for this study. However, the authors can mention and include basic analysis of one representative gt6 E2 structure, if time does not permit molecular dynamics simulations of such a structure to be included in this study.

We added text at line 463 to discuss the comparison of our models and the structures published by Tzarum et. al. We also performed pairwise RMSD analysis of our models with multiple E2 crystal structures, this is provided in S1 Table.

4. The Modeling methods section requires more details for readers to understand and potentially to utilize these methods in their own work. For instance, what versions of Modeller and Rosetta were used? It is noted that models were scored “using talaris2014”, and most readers may not understand that this is a scoring function in Rosetta. For reproducibility and clarity, the authors should provide the details of the command line parameters that were used for Modeller, LoopModel, FloppyTail, etc., for instance as reported by Adolf-Bryfogle et al. PLoS Comp. Biology 2018 14(4):e1006112 in their supporting information text.

We have amended the materials and methods in line with these suggestions (Line 684-689) and included the modelling scripts as S4 File.

5. The 1b09 E2 ectodomain model PDB, provided as a supplemental file, seems to have erroneous residues near the C-term, possibly an artifact from the x-ray structure. The C-terminal residues should be “NWT”, but instead they are “NIT”. It is suggested that, if this error is confirmed, that simulations be re-run, or at very least the authors should state in the text why it is inconsequential.

Thank you to the reviewer for pointing out this mistake. After careful consideration we decided not to rerun the simulations. However, we have provided a note on this error and justification for our decision at line 761.

**Minor comments:**

6. Line 130. It seems that E2 should be residues 384-746, not 384-747.

This has been amended.

7. Line 162. 1a53 and 1b09 E2 ectodomain structures were reported by Flyak et al., not just 1a53. Evidently the authors had a typo where 1b09 was replaced by 1a53 throughout the text, which they plan to correct, but in any case, they should note both isolates used in the set of structures reported by Flyak et al.

This has been included on line 164.

8. Lines 186-187. “...but again various studies have demonstrated conformational heterogeneity in these regions”. Citations should be given here.

References have been included.

9. Line 198. “unglycosylated” is not commonly used, “aglycosylated” should probably be used here for clarity.

This has been amended.

10. Lines 221-222. A “~30% amino acid divergence” is noted between the isolates studied here, and there is a reference to a multiple sequence alignment in Fig. S4. It would be helpful for readers to better understand the sequence divergence if the authors can include the actual pairwise sequence identities between E2 ectodomains for these three isolates (H77, J6, 1b09) in a table, optionally including the sequence % identities with and without HVR1.

We have revised S4 Fig in line with this suggestion.

11. Line 238. “in place of a side change” should be “in place of a side chain”.

This has been amended.

12. Lines 356-357. “...generate full-length models of E2; ... no known structural homologues of E2.” It seems that rather than “full-length” E2, the authors are only modeling E2 ectodomains, omitting the transmembrane regions, and also omitting the C-terminal “stem” regions of the ectodomains (aa 646-717). To avoid reader confusion, the authors should change this wording, and possibly clarify what is missing. Regarding the absence of “known structural homologues of E2”, there are several x-ray structures of E2 itself which the authors used directly to generate their models, as noted by the authors in the next sentence. The authors did need to model missing loops and the N- terminus, which can be considered a challenge, but having no known E2 structural homologues should not be stated as a challenge from a modeling standpoint.

This text has been reworded to avoid ambiguity (line 450).

13. Khan et al. (reference 14) previously reported SAXS analysis of several forms of the J6 E2 protein, but no comparison with those results was seen in the present study. Their SAXS results should be noted, and compared with the current SAXS results.

We have included a comparison of our data with that of Khan et. al. (line 404-417). Our data is very consistent with theirs, which increases confidence in our work.

14. It is not clear why the authors selected PDB code 6MEI rather than 6MEJ, as the latter structure includes more residues in HVR1 (starting at residue 405 rather than residue 410) and has slightly higher resolution (2.8 Angstroms). A note on the selection of this structure should be included in the Results or Methods.

A note on this is included (line 696).

**Reviewer #2:** The authors construct three different E2 ectodomain structures and use molecular dynamics (MD) simulations to compare their dynamic properties. The overlap in the behavior of these ectodomains generally support their findings. As HVR2 is not hypervariable in genotype 2 it would of interest to extend the analysis of HVR2 by comparing J6 with the two genotype 1 isolates to see whether differences can be identified.

We analysed alignments of GT1 and GT2 HVR-2 for sequence diversity and observed equivalent sequence divergence (25-30%) in both genotypes. Therefore, we do not believe further focus on HVR-2 is appropriate. The HVR-2 alignments used for this analysis can be provided if necessary.

The authors apply a careful and methodical MD approach to increase our very limited understanding of HCV envelope protein E2 structure and function. The work appears to adhere to current MD standards and is performed in a clear fashion. It is nice that the authors provide SAXS evidence in support of their MD work. In addition, the discussion is relevant and well-balanced with a few exceptions listed

1. The potential contribution from missing HCV envelope proteins elements, most notably E1, seems skewed. Specifically, while the authors cite studies that suggest that HVR1 is on the surface of virions that does not, by itself, prove that it is not found there in an interaction with another envelope protein motif. Thus, if the interaction partner (e.g. E1) is missing then It does not seem overly strange that regions of E2, not constrained by adjacent secondary structure elements in E2 would be exhibiting high flexibility. While the authors interpretation of what their study means in terms of a disordered HVR1 is an intriguing one it seems the evidence for this is still lacking.

We broadly agree with the reviewer, but we would argue that we included a clear caveat to our work that discusses the limitations of using monomeric E2 and integrates the best current evidence for which regions are likely to buried in the E1E2 complex. Nonetheless we have added a further statement on line 632 to reassert this important limitation.

2. While the theory of a disordered HVR1 becoming ordered upon SR-BI engagement is intriguing it lacks support in the submitted manuscript. Partly due to the point made in bullet 1 on the reasons for HVR1 flexibility and partly because it assumes an HVR1-SR-BI interaction. However, HVR1-deleted sE2 can interact with SR-BI (Scarselli et al., 2002) if the right mutations are introduced and HVR1- deleted HCVcc can still depend on SR-BI during entry. This indicates that HVR1 is not the sole SR-BI interacting partner of E2 and perhaps suggests that other, indirect, effects of HVR1 deletion on SR-BI interaction are at play. Figure 9 seems particularly overreaching and it may even be considered misleading as the detailed atomic structures used to depict the hypothesis are not being used to establish the point being made (one would expect that atomic structures would be used to inform how and where the interaction occurs which is not the case). This is not to say that the findings regarding HVR1 movement correlating with changes throughout the E2 ectodomain (Figure 7) are not both compelling and extremely interesting, but rather it is a call to caution as to which interactions may precipitate this when considering full E1/E2 complexes on infectious particles interacting with a cell during entry.

On the matter of sE2 interacting with SR-B1 via HVR-1: the reviewer is referring to Fig. 3C of Scarselli et. al. in which delta HVR-1 sE2 with adaptive mutations (V514M and L615H) exhibit enhanced binding to HepG2 cells. In this particular experiment the binding of sE2 is likely to be multimodal (potentially also binding to attachment factors, such as HSPG), therefore it is not a good system for specifically examining E2-SR-B1 interactions. The cleanest system to study this is CHO cells expressing SR-B1; Scarselli et. al. use these cells to demonstrate complete loss of SR-B1 binding upon deletion of HVR-1 (Fig 8 of that paper). This observation has been recapitulated by two other groups (<http://www.jbc.org/content/287/42/35631.long>, <http://www.jbc.org/content/287/37/31242.long>). Therefore, we assert that the best evidence suggests that E2-SR-B1 interactions are HVR-1 dependent.

We accept that the proposed significance of E2-SR-B1 interaction is quite speculative and understand the reviewer’s concerns; therefore, we have reworded the text to soften this proposal (line 600) and have moved the associated figure to the supplementary material.

**Reviewer #3:** The manuscript by Stejskal et al. reports an analysis of the structural and dynamic properties of the E2 protein of the HCV virus. This protein is a key factor for HCV pathogenicity and is an important target for neutralizing MAbs. Characterizations of the E2 are also believed to hold important implications for the development of anti-HCV vaccines. Recent experimental investigations have provided an insightful but fragmentary picture of the protein structures. The main aim of this study is to complement available structural data with information derived by molecular dynamic simulations. Although MD studies on this system have been recently reported, the present study represent a significant advance in terms of simulation timescale, completeness of E2 structural model and covering of the strain variability of HCV. The work is technically sound and the data are clearly illustrated throughout the manuscript. The computational work is integrated by SAXS experiments that, although at low resolution, are in line with the MD results. On the basis of these considerations, I believe that the manuscript can be considered for publication. There are, however, some points that should be addressed:

1. The authors observed that E2 glycosylation has limited effects on the protein structure/dynamics. It is known, however, that E2 glycosylation, at least at specific sites, is essential for virus entrance and infectivity. The authors should comment this point.

We have amended the discussion to include this (line 500).

2. The authors correlated the observed flexibility of the different E2 regions with their sequence variability detected in CV strains. In this framework, how do the authors explain the rather strict sequence conservation of the AS412 epitope with its remarkable flexibility (Fig. 2) (see also Balasco et al Curr Med Chem. 2017 24(36):4081-4101 and Int J Biol Macromol. 2018, 116:620-632 for a discussion on this feature that is also observed in other HCV proteins).

Thank you for this suggestion. We have performed extensive new analysis of AS412 (see above) and have included a specific comment on this (line 537).

3. The authors performed a re-refinement of the PDB models before starting the MD. They found an improvement of the value of the crystallographic indicator R-factor (what about the R-free?). In my opinion this is very good a practice. The authors found a pattern of cis/trans peptide bonds that is closer to that observed in more recent E2 structures. Does this also reflect in a pair-wise analysis of the RMSD values of the core of the protein among these different E2 structures? I wonder whether the deposited models within the PDB should be amended to consider these improvements. Did the authors also try in their re-refinement process the RE_DO automated server?

Thank you to the reviewer for this positive comment. RMSD comparison of our refined models with multiple published E2 structures are now provided in S1 Table. We have also reported the Rfree values of our refined structures (line 661). We did not try the RE DO automated server as it is unable to automatically correct the cis/trans peptide bonds.
