# Supplementary material for: Flexibility and intrinsic disorder are conserved features of hepatitis C virus E2 glycoprotein
Source: PLoS Comput Biol. 2020 Feb 28;16(2):e1007710. doi: 10.1371/journal.pcbi.1007710 (PMC7065822; doi:10.1371/journal.pcbi.1007710)
Supplement: Attachment — Submitted filename: Detailed Response to Reviewers 30.01.20.docx [file pcbi.1007710.s022.docx]

Below we have listed the verbatim reviewers comments and our responses. For clarity, we have provided line number references for both the marked-up and clean (in parentheses) copies of the manuscript.

**Reviewer #1:**

- One minor comment is that the authors don't seem to specify the type of RMSD (backbone, C-alpha, or all-atom) used at several points, including the new Table S1 and the legend for Figure 5. The authors do, at one point, note that backbone atoms are used for RMSF calculations for the MD output in the Methods, so it is likely that they also use backbone atoms for RMSD calculations between structures. However, for clarity and to avoid ambiguity, the authors should specify in Table S1, Figure 5, and elsewhere as needed that backbone RMSDs are being used, or another type of RMSDs as warranted.

We agree that this should be clarified, thank you for pointing it out. We used the polypeptide backbone for all RMSD calculations. We have added references to this in the Results (Line 178 [178]), Materials and Methods (Line 666 [660]) and the appropriate figure legends (Lines 1031 [1025], 1040 [1034], 1046 [1040], 1108 [1102] and 1122 [1116]).

**Reviewer #2:**

- This is likely because the authors did not stratify their analysis to subtypes. In fact, HVR2 of gt 2a is quite different from HVR2 of gt 2b, but they are both quite conserved within-subtype. Similarly, gt 1a is fairly conserved, whereas gt 1b has a high degree of sequence diversity, particularly in the C-terminal part of HVR2. I think these differences are of interest and warrant further investigation, but I do not consider it critical for the current manuscript.

We would like to thank the reviewer for this correction and clarification, we had not appreciated the differences in subtype specific variations in HVR-2. We agree that it would be interesting to integrate this information in to future investigations.

- Firstly, there is no added statement at line 632, which is in the Methods section of the paper. I assume that the lines referred to 600-601? Secondly, the adjustment line added is missing the point I was raising. While I do appreciate the caveat present in the initial submission it is not simply a matter of a region being either buried or exposed on E1E2 (on particles) or soluble E2, respectively. Rather, a region can be at the surface of a protein without being highly flexible. Thus, flexibility of HVR1 and AS412 on soluble E2 are not necessarily evidence of flexibility in higher order structures in the context of E1E2. This is the point I think it would be good to make explicitly.

Firstly, the disparity in line numbers is due to the fact that our previous response referred to the marked-up (not clean) version of the text. We have included references to both documents in this response. Thank you to the reviewer for clarification about this legitimate concern. We have added a further statement on line 601 (595), such that our final caveat reads: “However, without a detailed structure of E1E2, we cannot be certain which regions are buried within the complex, or predict whether apparently flexible regions of E2 are stabilised in the context of the E1E2 oligomer.”

- Firstly, line 600 is not the place where adjustments were made. Are the authors referring to line 575-580? Also, there appears to be a comment associated with this section. Secondly, I fully agree that evidence exists for an SR-BI interaction with HVR1. However, evidence is also abundant that it is much more complicated than a “simple” interaction (for which the two papers highlighted in the author response are good examples) and could involve many other “partners” both within and outside the envelope proteins themselves. In the authors response they write that the interaction is “HVR1-dependent”, which is very different from writing that the interactions occur via HVR1 as they do in the Introduction. Both could be true and I do not have any issue with the speculations on what the significance of the MD simulations could be in terms of potential SR-BI interactions with HVR1, as long as these limitations are clear. This is particularly important when the authors had a figure in the main article showing this interaction using high resolution molecular structures in the absence of these important caveats. Moving it to the supplementary was a good idea.

We appreciate the reviewers concerns about the potentially complex nature of HCV-SR-B1 interactions and, therefore, have added further text (and associated citations) on line 568 (566). This now reads: “Whilst HCV-SR-B1 interactions may be complex in nature [25,70,71], HVR-1 is the putative E2-SR-B1 binding site [17,22–24], hence, we hypothesise that during virus entry HVR-1 transitions from a disordered and mobile state to one in which it is constrained in the E2-SR-B1 interface (S8 Fig).”

**Reviewer #3:**

In some places crystal is misspelled as crytsal.

Thank you for pointing this out. These have been corrected (lines 305 [305] and 312 [311])
